# The prognostic value of lncRNA SNHG6 in cancer patients

**DOI:** 10.1186/s12935-020-01383-9

**Published:** 2020-07-06

**Authors:** Haixiang Shen, Qiwang Mo, Xin Xu, Ben Liu

**Affiliations:** 1 Department of Urology, First Affiliated Hospital, School of Medicine, Zhejiang University, Qingchun Road 79, Hangzhou, 310003 Zhejiang China; 2 Department of Urology, Shengzhou People’s Hospital, Shengzhou, 312400 Zhejiang China

**Keywords:** Cancer, Prognosis, Long no-coding RNA, SNHG6

## Abstract

**Background:**

Although tremendous improvement has been seen in cancer diagnosis and treatment, its morbidity and mortality is still high due to lack of ideal biomarkers. An increasing number of studies have demonstrated that the expression of lncRNA small nucleolar RNA host gene 6 (SNHG6) has significantly negative correlation with various cancer prognosis. The present meta-analysis was aimed to clarify the potential of clinical application of SNHG6 in cancers.

**Methods:**

A detailed literature review was conducted by searching through PubMed and Web of Science databases. The expression level of SNHG6, clinicopathological features and survival outcomes were extracted from eligible studies. Pooled analysis was performed with a DerSimonian-Laird random-effect model. The results were further validated through the Cancer Genome Atlas (TCGA) dataset.

**Results:**

Five studies with a total of 487 cases were finally included in this meta-analysis. The results demonstrated that a high expression of SNHG6 was significantly associated with an increased risk of poor overall survival (OS) in cancer patients (HR = 2.06, 95% CI 1.56–2.73). Similar results from the TCGA dataset further confirmed our findings.

**Conclusions:**

Overexpressed SNHG6 was significantly associated with poor prognosis in various cancers. Therefore, SNHG6 may become a novel molecular target for treatment and prognostic evaluation.

## Background

Cancer has become the leading cause of death globally. Over the past decades, although tremendous improvement has been achieved in its diagnosis and treatment, the prognosis is still poor, especially for advanced cancers [1]. In the United States, it was estimated that approximately 1,762,450 cancer cases would be diagnosed and an estimated 606,880 people would die from cancer in 2019 [2]. It can impose huge financial burden on patients’ families and society. Therefore, novel biomarkers are urgent to be discovered for early diagnosis, treatment and prognostic assessment.

Among ~ 90% of human genome DNA that are transcribed, only 2% of them encode protein. The others which encode non-protein are named as non-coding RNAs (ncRNAs) [3, 4]. Long non-coding RNAs (lncRNAs) is a category of endogenous ncRNAs with a length of more than 200 nucleotides, which accounting for > 70% of ncRNAs [5–7]. Increasing studies have demonstrated that lncRNAs is involved in various normal cellular processes including development, differentiation and metabolism by epigenetic regulation, transcription and post-transcriptional regulation [8–11]. Recently, dysregulation of lncRNAs has been reported to be associated with oncogenesis and cancer progression [12–15], which suggested the potential of lncRNAs to be a new biomarker for early diagnosis, prognostic value and therapeutic target.

Small nucleolar RNA host gene 6 (SNHG6), also known as U87HG, is a novel lncRNA located in chromosome 8q13.1. In 2016, Chang et al. firstly illustrated that SNHG6 was overexpressed in hepatocellular carcinoma (HCC) and promoted tumor growth and metastasis by inducing epithelial to mesenchymal transition (EMT) [16]. In recent years, accumulating evidence revealed that SNHG6 was aberrantly expression in various types of cancers and was significantly correlated with clinical stage and prognosis [16–18]. Whereas, due to small sample size in these researches, the prognostic value of SNHG6 is limited and controversial. Therefore, this meta-analysis was conducted to investigate the potential prognostic value of SNHG6 in human cancers.

## Methods

### Literature search

This study was performed according to the Preferred Reporting Items for Systematic Reviews and Meta-Analyses (PRISMA) statement [19]. Studies on the association between SNHG6 expression and prognosis of human cancers were identified from PubMed and Web of Science (WOS) database (up to October 30, 2019) with the following search strategy: (“small nuclear RNA host gene 6” or “SNHG6”) and (“cancer” or “carcinoma” or “neoplasm” or “tumor”). Reference lists of the identified articles and relevant reviews were manually examined for additional eligible studies. Potential eligible studies were selected by two independent authors (XX and HXS), and controversial articles were resolved by discussion and consensus.

### Inclusion criteria

Studies included in this meta-analysis met all the following criteria: (i) study population was cancer patients; (ii) OS were analyzed according to SNHG6 expression pattern; (iii) multi-adjusted hazard ratios (HRs) and their 95% confidence intervals (CIs) were provided; (iv) articles were published in English or Chinese. Studies only reported risk estimates from univariate analysis were excluded from this meta-analysis.

### Data extraction

The following information was extracted from each included study by two independent authors (XX and HXS): the surname of first author, publication year, country, number of patients, age of patients, methods to determine SNHG6 expression, cut-off value, prognostic data, and adjusted variables. Any discrepancies were solved by consensus.

### Quality assessment

The methodological quality of each included study was evaluated by two independent reviewers (XX and HXS) using the Newcastle-Ottawa Scale (NOS) with reasonable modifications. NOS is an eight-item instrument that focused on the characteristics of study population, study comparability, follow-up and outcome of interest. The total score of NOS is 9. A study with a score of ≥ 7 was considered to be of high-quality.

### Validation by reviewing public data

This study meets the publication guidelines provided by The Cancer Genome Atlas (TCGA). Gene Expression Profiling Interactive Analysis (GEPIA) [20] was used to verify the correlation between SNHG6 and OS and to assess the expression pattern of SNHG6 in human cancers.

### RNA extraction and qRT-PCR

Total RNA was extracted from the human RCC cell lines 786-O and Caki-1, as well as one normal kidney cell line HK-2 using the RNAiso Plus (TaKaRa, Japan). Then the RNA was transcribed into cDNA using the PrimeScript RT Reagent Kit (TaKaRa, Japan). qRT-PCR assay was performed using ABI 7500 FAST Real-Time PCR System (Applied Biosystems, USA) and SYBR Green PCR Kit (Takara, China). The mRNA expression level was calculated using the 2^−∆∆Ct^ method after normalization with β-actin. The primers involved were SNHG6 Forward 5′-ATACTTCTGCTTCGTTACCT-3′; Reverse 5′-CTCATTTTCATCATTTGCT-3′; β-actin Forward 5′-ATCATGAAGTGTGACGTGGAC-3′; Reverse 5′-GACTCGTCATACTCCTGCTTG-3′.

### Statistical methods

The impact of SNHG6 expression on the OS of cancer patients was quantified by the summary HRs and their 95% CIs. A DerSimonian-Laird random-effect model [21] was used to calculate the summary risk estimates. Existence of heterogeneity among included studies was determined using the Q statistic (significant level set at 0.1) [22]. I^2^ statistic was further used to assess the degree of heterogeneity (low heterogeneity: I^2^ < 25%; moderate heterogeneity: I^2^ = 25–50%; high heterogeneity: I^2^ > 50%). Sensitivity analysis was performed by sequential omission of each included study. Publication bias was assessed using a visual funnel plot. All of the statistical analyses were performed with STATA 11.0 (StataCorp, College Station, Texas USA), using two-sided P values.

## Results

### Literature search and study characteristics

**Fig. 1:**
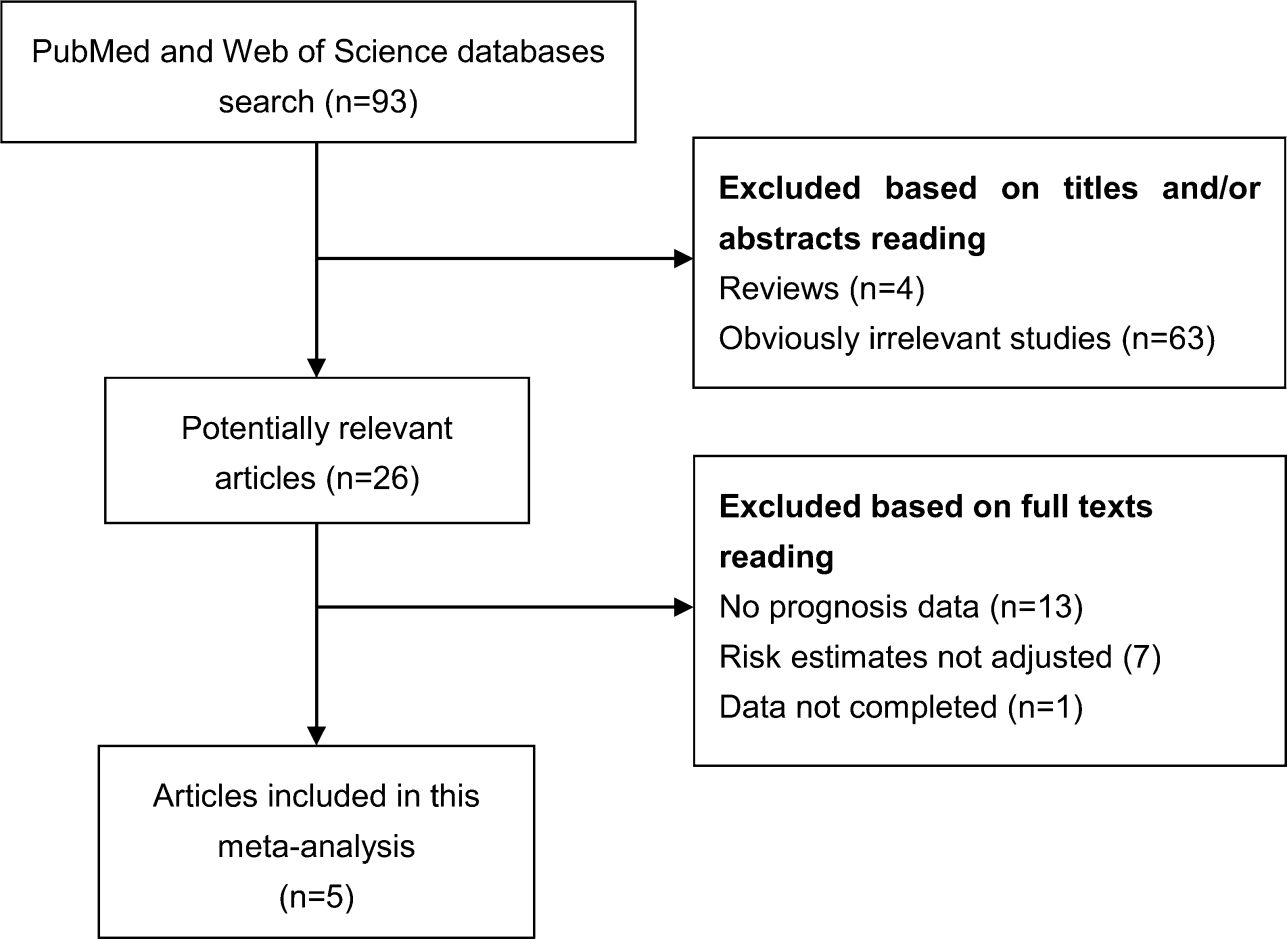
The diagram shows the procedure of literature search and study selection

Literature search and selection has been shown in Fig. 1. Five studies [23–27] were finally included in this meta-analysis aimed to evaluate the association between SNHG6 expression and OS of cancer patients. Three studies were performed in colorectal cancer (CRC), one in renal cell carcinoma (RCC) and one in glioma. All of these studies were published between 2018 and 2019. These studies were performed in China and involved a total of 487 cases. SNHG6 expression data was obtained by real time PCR (RT-PCR). The methodological quality, as assessed by the NOS, ranged from 8 to 9 (with a mean of 8.4). The main characteristics of each study have been summarized in Table 1.

**Table 1:** Main characteristics of the studies included in the meta-analysis

Authors	Year	Cancer	HR (95% CI)	Method	No.	Follow	NOS
An et al.	2018	RCC	1.93 (1.23–3.05)	RT-PCR	81	80 m	9
Li et al.	2018	CRC	2.57 (1.06–6.25)	RT-PCR	74	NR	8
Meng et al.	2018	Glioma	1.65 (1.10–3.32)	RT-PCR	71	60 m	9
Xu et al.	2019	CRC	2.48 (1.60–5.86)	RT-PCR	120	NR	8
Yu et al.	2019	CRC	2.83 (1.20–8.36)	RT-PCR	141	NR	8

*RCC* renal cell carcinoma, *CRC* colorectal cancer, *HR* hazard ratio, *95% CI* 95% confidence interval, *NR* no report

### Systematic review

Three studies performed in CRC included 74, 120, and 141 subjects, respectively. The study by Li et al. [24] indicated that SNHG6 was generally up-regulated in CRC tissues and high level of SNHG6 expression was strongly associated with advanced tumor stage (P = 0.026) and poor prognosis (P = 0.0215). The study by Xu et al. [26] reported that SNHG6 expression was an independent prognostic biomarker (HR = 2.48, 95% CI = 1.60–5.86, P = 0.002) for CRC in the multivariate analysis. Yu et al. [27] found high expression of SNHG6 was positively related with tumor size, advanced TNM stage, and tumor metastasis. In addition, SNHG6 was an independent prognostic factor of poor OS (HR = 2.83, 95% CI 1.20–8.36, P = 0.018) and RFS (HR = 2.07, 95% CI 1.17–6.20, P = 0.020). The study by An et al. [23] reported that elevated SNHG6 was significantly associated with tumor progression and lymph node metastasis in a total of 81 cases of RCC. In addition, SNHG6 expression was associated with overall prognosis in RCC. Meng et al. [25] investigated the role of SNHG6 in glioma and found that the expression of SNHG6 was negatively associated with the OS (HR = 1.65; 95% CI 1.10–3.32; P = 0.0076).

### Overall analysis

**Fig. 2:**
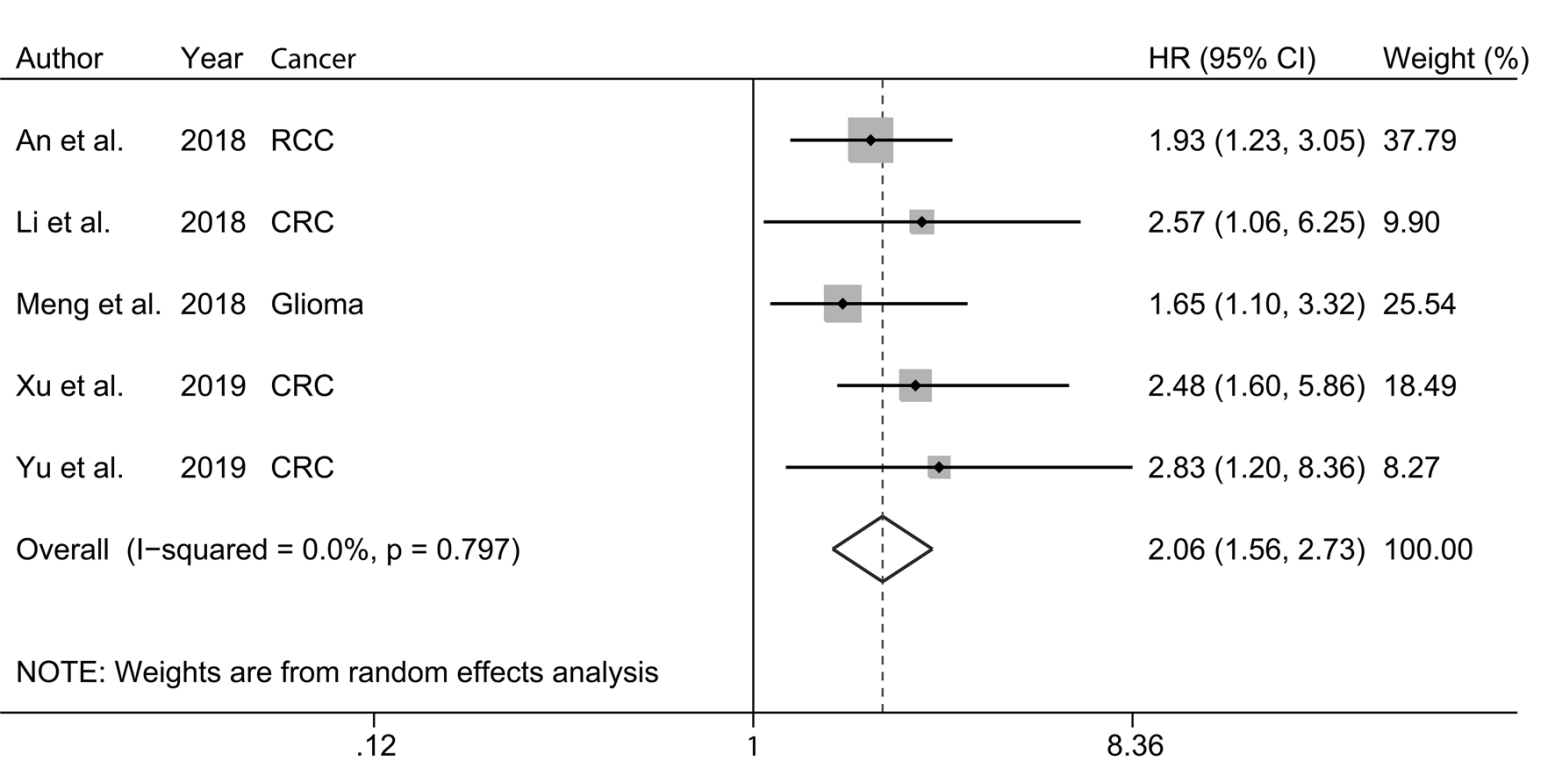
Forest plot for the correlation between expression level of SNHG6 and overall survival (OS)

The HRs for each included study and for the combination of all studies are shown in Fig. 2. A high expression of SNHG6 was significantly associated with an increased risk of poor OS (HR = 2.06, 95% CI 1.56–2.73). There was no obvious heterogeneity among the studies (I^2^ = 0.0%; P = 0.797).

### Sensitivity analysis and publication bias

**Fig. 3:**
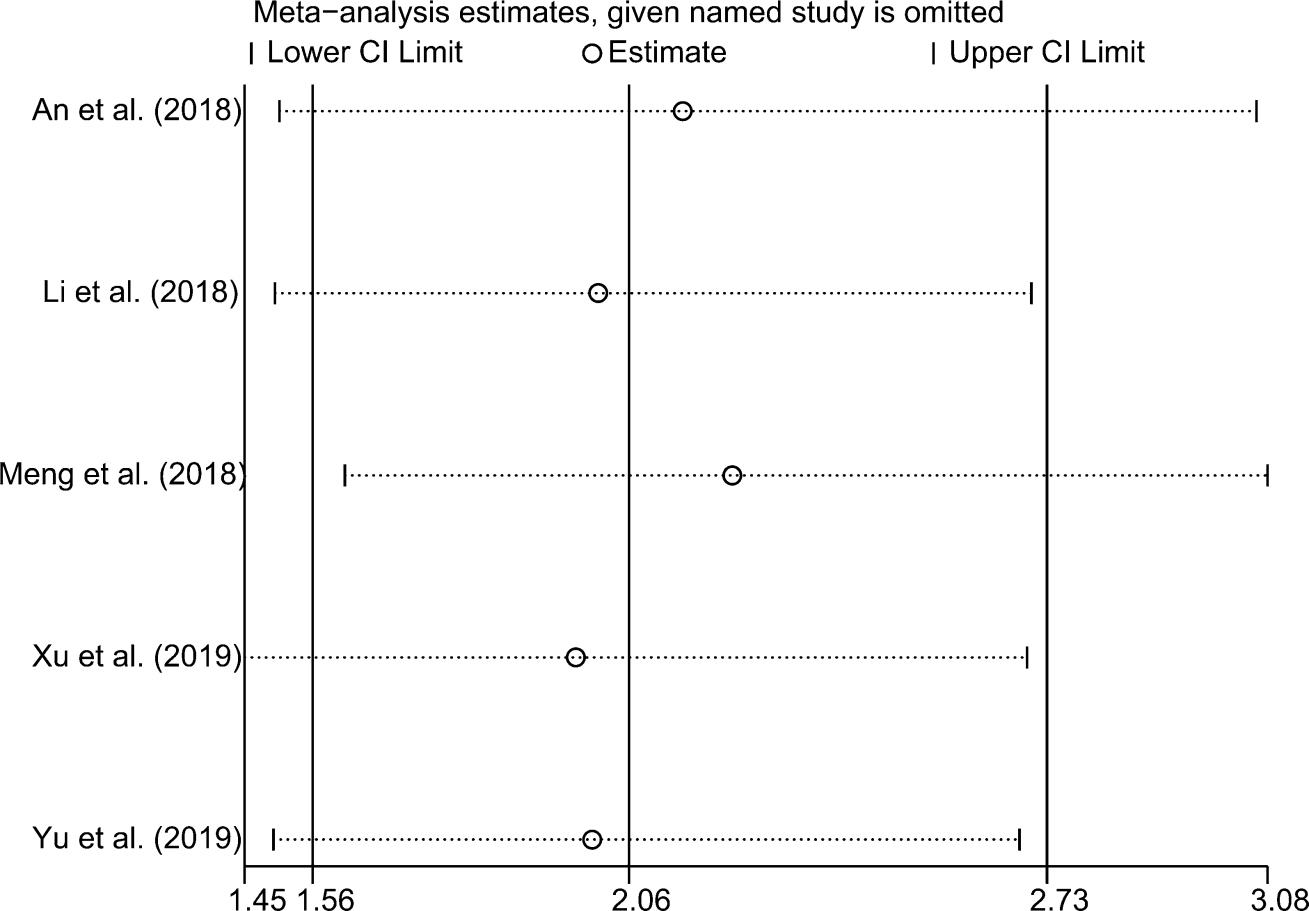
Sensitivity analysis of the included studies concerning SNHG6 and overall survival (OS)

**Fig. 4:**
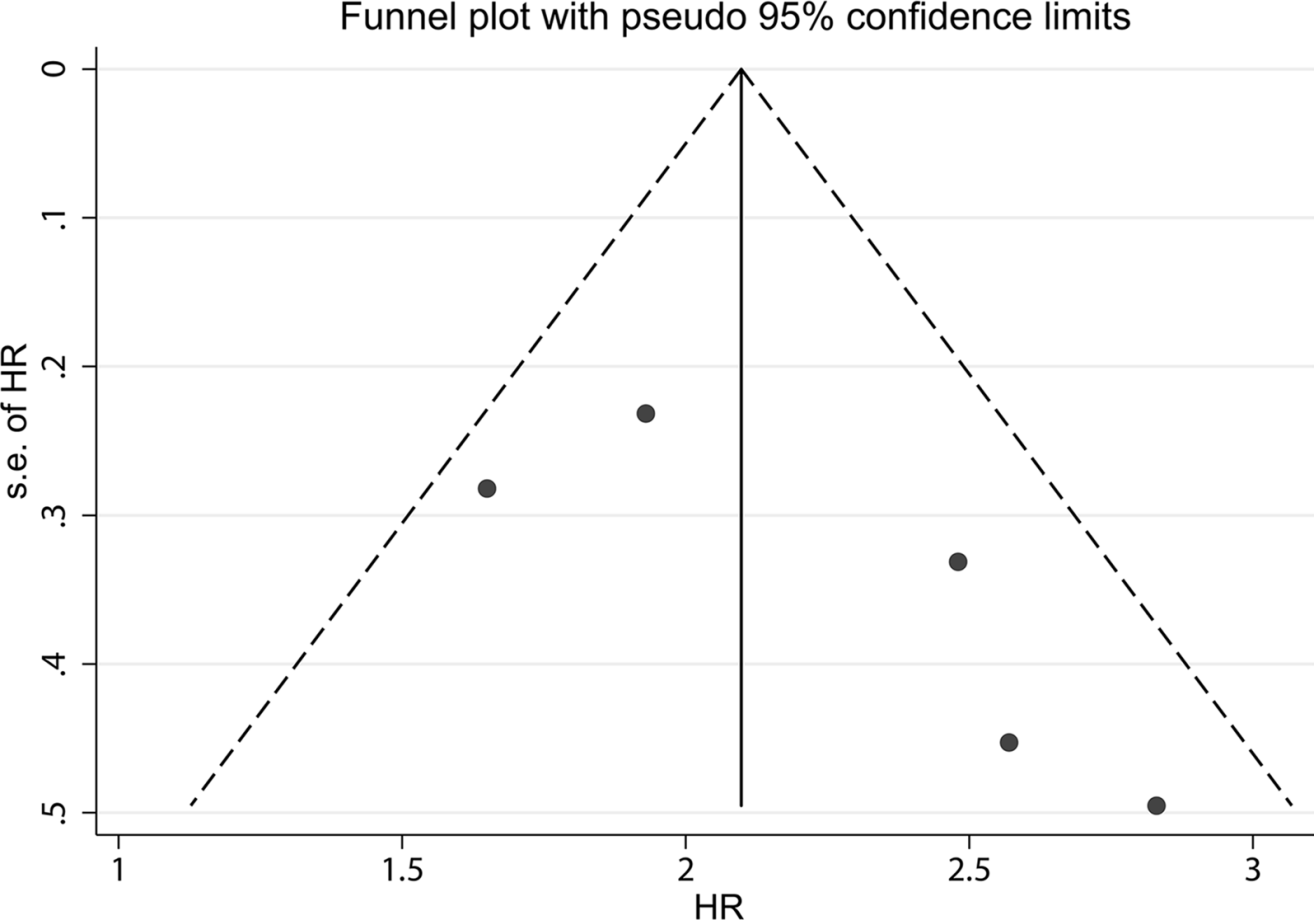
Funnel plot of SNHG6 for overall survival

In order to assess the stability of the pooled risk estimate of the association between SNHG6 expression and OS, sensitivity analysis was performed by omitting each included study in turn. As shown in Fig. 3, the pooled result was not dominated by any single study. There was no evidence of publication bias with a visual funnel plot (Fig. 4).

### SNHG6 and OS of CRC

Three studies reported data on the relationship between SNHG6 expression and OS of CRC. The summary data based on these studies indicated that SNHG6 status was significantly associated with the OS with a combined HR of 2.58 (95% CI 1.63–4.09). No obvious heterogeneity was observed across studies (I^2^ = 0.0%, P = 0.976).

### Validation of the SNHG6 expression in RCC cells

The mRNA expression of SNHG6 in two RCC cell lines (786-O and Caki-1) and one normal kidney cell line (HK-2) was detected using qRT-PCR. As a result, the expression of SNHG6 in RCCs was significantly upregulated compared with that in HK-2 cells (Additional file 1: Figure S1).

### Validation of the results in TCGA dataset

**Fig. 5:**
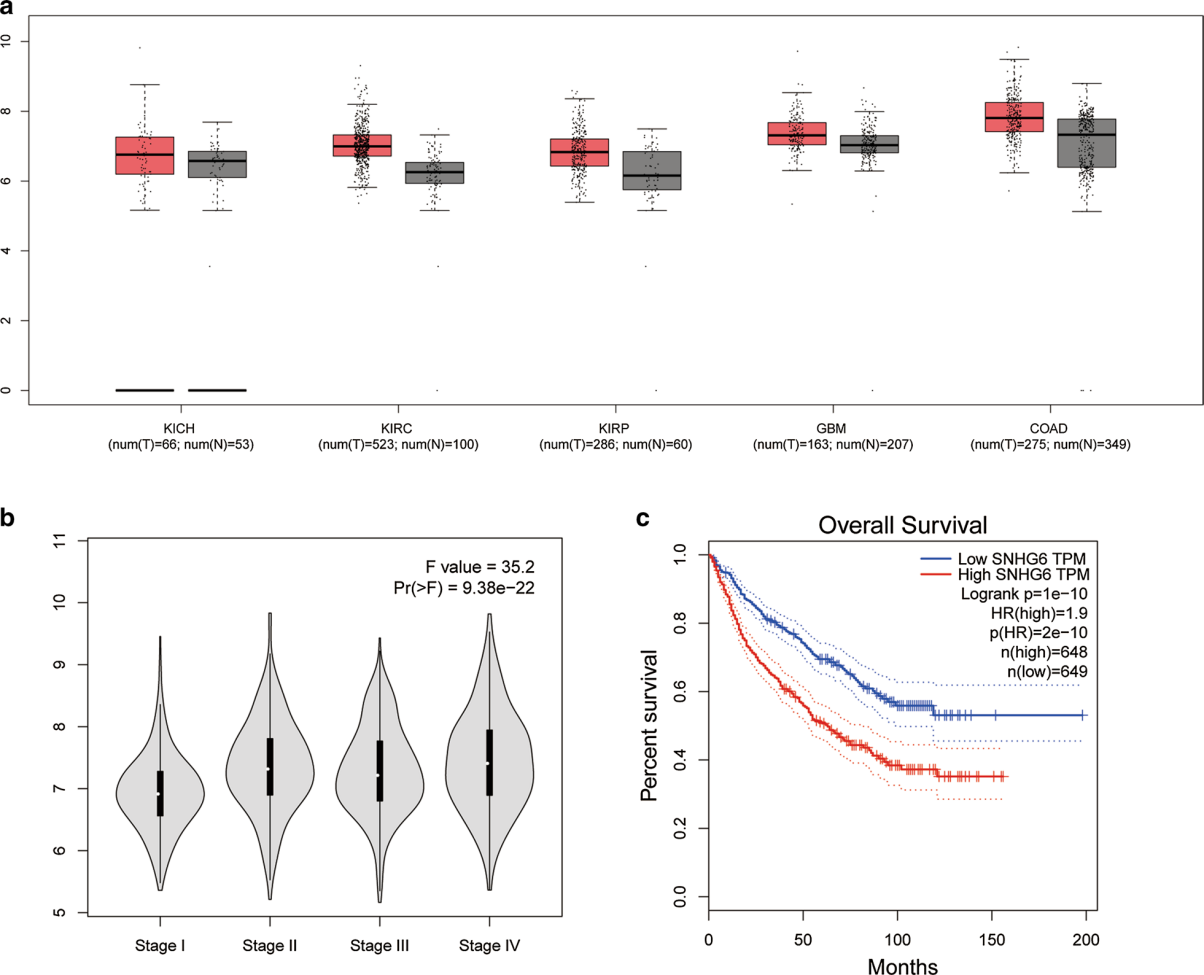
Validation of SNHG6 expression of various cancers in the TCGA dataset. **a** The expression levels of SNHG6 in KICH, KIRC, KIRP, GBM and COAD. **b** Violin plot showing that SNHG6 expression was significantly associated with clinical stage in these human cancers. **c** Overall survival plot of SNHG16 in TCGA cohort (n = 1297, log-rank *p *< 0.001)

We further used TCGA dataset to investigate SNHG6 expression level in human cancers. As shown in Fig. 5a, SNHG6 was upregulated in kidney chromophobe (KICH), kidney renal clear cell carcinoma (KIRC), kidney renal papillary cell carcinoma (KIRP), glioblastoma multiforme (GBM) and colon adenocarcinoma (COAD) when compared with non-cancer tissues. The violin plot indicated that SNHG6 expression was significantly associated with clinical stage in these human cancers (Fig. 5b). Finally, a survival plot merging SNHG6 expression data and OS data of RCC, GBM and CRC from the TCGA dataset were performed. As shown in Fig. 5c, the overexpression of SNHG6 was significantly associated with an unfavorable OS, which was consistent with our results in this meta-analysis.

## Discussion

In the past decades, a large amount of lncRNA transcripts were discovered by high throughput genome sequencing technologies. Along with the emerging evidence, the pivotal role of lncRNAs in oncogenesis was unveiled gradually [28, 29]. lncRNAs, including SNHG6, were also indicated as prognostic biomarkers in literature [23, 24]. However, the prognostic value of SNHG6 was limited and contentious due to the small sample size. Therefore, our study aimed to clarify the potential prognostic value in multiple cancers through a pooled analysis.

As a novel lncRNA, SNHG6 had been discovered overexpressed in various types of cancers, including CRC [30], HCC [16], breast cancer [31], gastric cancer [32], lung cancer [33], glioma [25] and osteosarcoma [18]. In addition, a negative correlation between SNHG6 expression and prognosis has been demonstrated in the literature [33, 34]. As for the carcinogenesis role of SNHG6, the underlying molecular mechanisms had been partly elucidated. Through the competing endogenous RNA (ceRNA) mechanism, SNHG6 can competitively sponge miRNAs and regulate their target genes. In 2016, Chang et al. firstly reported that SNHG6 facilitated tumor growth and metastasis in hepatocellular carcinoma by competitively binding miR-101-3p to regulate ZEB1 [16]. Wang et al. found that the up-regulation of SNHG6 significantly repressed the expression of miR-125b and increased the NUAK1 expression [35]. miR-26a-5p/ULK1 and miR-26a-5p/MAPK6 axis were also regulated by SNHG6 and participated in the development and progression of breast cancer and osteosarcoma, respectively [18, 31]. Another study by Jafari-Oliayi et al. [34] also found that the expression of SNHG6 was significantly upregulated in primary breast cancers. SNHG6 silencing led to G1 cell cycle arrest and suppressed cell proliferation. Several signaling pathways can be activated by SNHG6, including the MAPK and JNK pathway in gastric cancer [32], PI3K/AKT/mTOR pathway and TGF-β/Smad pathway in colorectal cancer [36, 37]. SNHG6 could also regulate cell cycle through interacting with crucial factors like p21 [32, 38]. In summary, elevated SNHG6 plays a vital role in cancer cell proliferation, migration and invasion.

In this meta-analysis, five original studies with a total of 487 cases were finally included and the results were pooled with adjustment for multiple confounding factors. The overall analysis showed that a high expression of SNHG6 was significantly correlated with an unfavorable OS (HR = 2.06, 95% CI 1.56–2.73). Moreover, the results of subgroup analysis were also demonstrated the negative correlation between expression level of SNHG6 and prognosis, which was consistent with overall analysis.

Several limitations of our study should be acknowledged. First, because we only included studies providing multi-adjusted results, the number of papers included in final analysis was relatively small. Secondly, all of the five included studies were performed in China with Chinese population. Therefore, the generalization of our findings is relatively limited. Thirdly, various therapies for different patients may have been used in included studies, which can lead to some bias. Finally, the definition for high SNHG6 expression was obscure and might be different in the included studies, which may also affect the final pooled results.

## Conclusion

In summary, the results of this meta-analysis support that SNHG6 overexpression is significantly associated with a poor prognosis in human cancers. SNHG6 may become a novel molecular target for treatment and prognostic evaluation. However, due to the limitations of our study, more well-designed studies are warranted to validate the role of SNHG6 in human cancers.

## Supplementary information

**Additional file 1: Figure S1.** The mRNA expression of SNHG6 in two RCC cell lines (786-O and Caki-1) and one normal kidney cell line (HK-2) was detected by qRT-PCR.

## Data Availability

All data and materials analyzed in this study are included in this published article.

## Abbreviations

lncRNA: Long non-coding RNA
WHO: World Health Organization
HCC: Hepatocellular carcinoma
EMT: Epithelial to mesenchymal transition
HR: Hazard ratio
CI: Confidence interval
OS: Overall survival
TCGA: The Cancer Genome Atlas
NOS: Newcastle–Ottawa Scale
CRC: Colorectal cancer
RCC: Renal cell carcinoma
KICH: Kidney chromophobe
KIRC: Kidney renal clear cell carcinoma
KIRP: Kidney renal papillary cell carcinoma
GBM: Glioblastoma multiforme
COAD: Colon adenocarcinoma
ceRNA: Competing endogenous RNA
