# Supplementary figures and images for: The prognostic value of lncRNA SNHG6 in cancer patients
Source: Cancer Cell Int. 2020 Jul 6;20:286. doi: 10.1186/s12935-020-01383-9 (PMC7339569; doi:10.1186/s12935-020-01383-9)

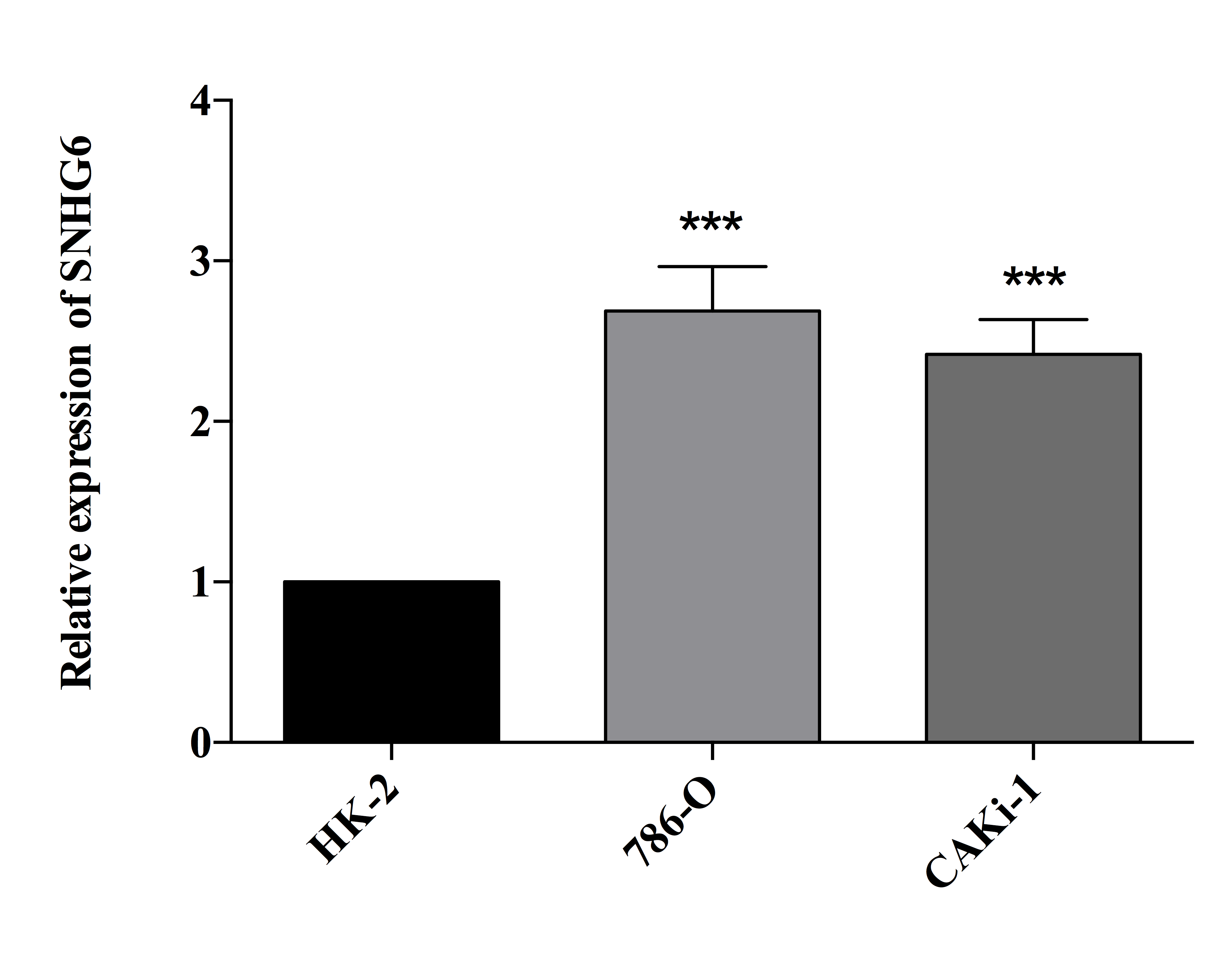

Supplement: Supplementary file 1 — Additional file 1: Figure S1. The mRNA expression of SNHG6 in two RCC cell lines (786-O and Caki-1) and one normal kidney cell line (HK-2) was detected by qRT-PCR. [file 12935_2020_1383_MOESM1_ESM.tiff]
